# Interactive effects of pulmonary pathologies and ventilation modes driving heterogeneous and anisotropic regional strain mechanics

**DOI:** 10.1038/s41598-025-27146-y

**Published:** 2025-12-02

**Authors:** Talyah M. Nelson, Kathrine A.M. Quiros, Mona Eskandari

**Affiliations:** 1 Department of Mechanical Engineering, University of California, Riverside, CA USA; 2 BREATHE Center, School of Medicine, University of California, Riverside, CA USA

**Keywords:** Multiscale biomechanics, Digital image correlation, Lung mechanics, Fibrosis, Emphysema, Positive pressure ventilation, Negative pressure ventilation, Heterogeneity, Anisotropy, Mechanical engineering, Asthma, Chronic obstructive pulmonary disease, Respiration, Biological physics

## Abstract

Pulmonary diseases are wide-spread, incurable, and commonly necessitate ventilatory intervention, which can lead to unintended ventilator induced lung injuries (VILI). Modern clinical devices utilizing positive pressure ventilation (PPV) may overdistend lung regions and initiate VILI compared to physiologically-analogous negative pressure ventilation (NPV) devices. Why this is the case remains to be fully understood, as studies of PPV versus NPV modes are scarce, particularly for lungs under pathological states. To address this major shortcoming, murine emphysematous or fibrotic lungs are inflated via a custom-designed electromechanical device capable of imposing PPV and NPV modes; digital image correlation simultaneously captures continuous local mechanical strains. While previously unattainable, here we couple traditional bulk pressure-volume lung analyses to local mechanics to discern potential VILI mechanisms interdependent on both ventilation mode (e.g. PPV and NPV) and pathological state (e.g. healthy, emphysematous, fibrosis). For all healthy and diseased groups, PPV-inflated lungs have significantly greater strain than NPV at low- to mid-inspiration volumes, indicative of local overdistention. Interestingly, at peak inflation, NPV strains are more prominent, and this may arise due to greater engagement of supplementary recruited regions. Deformation heterogeneities, anisotropy, and shear strains are most prominent for fibrotic, PPV-inflated lungs, indicating that the damaging regional imbalances of fibrosis are likely amplified by PPV. Additionally, when emphysema-weakened lungs are combined with PPV, central overdistensions occur, highlighting specific injury-prone areas. These local measures in pathological lungs expose pathways to VILI in PPV versus NPV, and insights can be used to inform clinical strategies, future research, and computational models.

## Introduction

Pulmonary diseases are an incurable and ever-present health threat, with rising cases as a result of rampant risk factors, such as environmental pollutants and smoking^1,2^. Chronic endurance of pulmonary diseases often necessitate clinical ventilation to support breathing, however, this frequently causes ventilator induced lung injury (VILI), which can be irreversible and even fatal, especially for lungs in a delicate pathological state^3,4^. Artificial modern ventilators primarily operate via positive pressure ventilation (PPV), where air is pushed directly into the trachea and upper lung regions^5^. In physiological breathing however, the diaphragm decreases lung internal pressure (relative to atmospheric) to draw air into the lung^5^. Negative pressure ventilation (NPV) devices mimic this, but are uncommon due to medical practicality purposes^6^. The deviation from physiological breathing mechanics with use of PPV has been shown to be a driving factor for destructive VILI^5,7,8^. However, this is not yet well-understood, as existing lung research compares PPV and NPV via standard global measures (e.g., pressure-volume, impedance, force expiration, etc.)—and lacks direct assessment of the local deformation patterns, which can cause VILI, such as regional strain heterogeneity^4,9–14^. Studies employing regional stretch measures are therefore necessary to rigorously assess the efficacy of PPV and NPV modalities. Furthermore and most notably, local analyses of PPV and NPV do not yet exist for cases of pulmonary disease—the impetus for ventilatory support—and yet such studies are irreplaceable, as localized disease manifestations likely render lungs even more injury-prone and further amplify differences between PPV and NPV^7,15^.

Herein, established dust- and elastase-exposure protocols are employed to model pulmonary fibrosis and emphysema, respectively, to assess the local PPV versus NPV response in pathology induced murine lungs for the first time^16,17^. A unique electromechanical dual-piston ventilator is used to inflate the healthy and pathological lung specimens, each with PPV and NPV, permitting robust comparative mechanical evaluations between the two ventilation modes on same lung specimens to minimize confounding factors^6,18^; concurrent to global pressure-volume measurements, digital image correlation (DIC) technology collects continuous, full-field, deformation measures^19^. This methodology—previously established for non-pathological states^5^, and now employed to consider disease mechanics—uniquely allows us to quantify lung surface strains as well as their heterogeneous, anisotropic, and evolutionary properties over the inflation profile^12–14,20–24^. Such insights can yield insights to VILI mechanics in PPV versus NPV, and ultimately advance fundamental comprehension of lung mechanics to inform clinical practices, future research, and computational models^25–29^.

## Methods

Twenty C57BL/6 J mice (male, 8–12 weeks) were procured from Jackson Laboratory (Bar Harbor, ME, USA) to conduct joint DIC-ventilation experimental protocols under approval from the University of California Riverside’s Institutional Animal Care and Use Committee (IACUC; protocol AUP#20200014). All methods were carried out in accordance with the institutional guidelines and regulations and are reported in accordance with ARRIVE guidelines. To induce disease states, the following exposure protocol, where these mice were also utilized in past studies ^12,15,13,30, was implemented^: five mice were intranasally exposed to agricultural dust (12.5%, 50 µl) thrice weekly for 21 weeks, per an established protocol wherein long-term peribronchiolar fibrosis ultimately manifests within the tissue^16^ (verified previously via Ashcroft’s score^31)^. Additionally, five mice were intranasally exposed to porcine pancreatic elastase (single dose, 0.9 µl) per the classic emphysema model, wherein tissue significantly degrades after 4 weeks^17^. Disease manifestation was confirmed, as detailed previously^15^. Age-matched controls (five mice per group) were administered 1X phosphate-buffered saline (PBS) under analogous dosage vehicles, frequencies, and durations to the respective fibrotic and emphysematous exposed mice.

We specify that the final sizes for each of the four groups (fibrotic exposed, fibrotic control, emphysematous exposed, emphysematous control) were *n* = 3–4, as specimens were eliminated due to leaking: a common occurrence in ex-vivo lung testing^32,33^. During the exposure periods, mice were housed at the University of California Riverside vivarium in micro-isolator cages with free-feeding, 12:12 h light-dark cycles, continuous physiological and behavioral monitoring, and weekly weigh-ins. Final fibrotic group weights were 31.5 ± 2.9 and 30.9 ± 4.5 g (exposed and control, respectively; post 21 weeks), and 28.0 ± 1.6 and 26.8 ± 1.4 g for the emphysematous group (exposed and control, respectively; post 4 weeks).

**Fig. 1:**
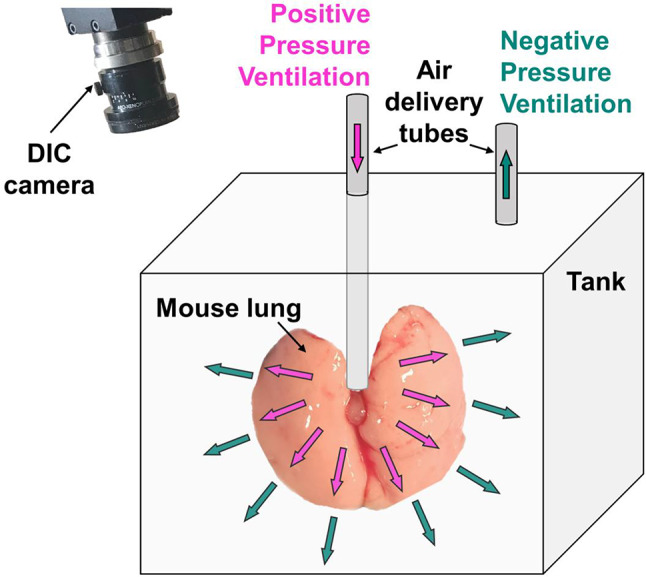
Schematic of positive and negative pressure ventilation applied to a murine lung: a custom electromechanical system permitted real-time global pressure and volume assessment, while local strains were collected via overhead DIC deformation imaging.

Following the exposure periods, mice were anesthetized and sacrificed via intranasal exposure to an isoflurane-doused cotton ball (5 ml), with additional cervical dislocation and bilateral thoracotomy. Lung-heart blocs were extracted, followed by tracheal cannulation and inflation to 0.5 ml via syringe to prevent atelectasis. Then, specimens were prepared for concurrent ventilation and DIC imaging (Fig. 1), as detailed extensively previously^5,12,13,20,21^. Succinctly, lungs were speckled and placed within the tank (partially filled with 1X PBS to maintain moisture and mitigate lung sliding friction) of our custom dual-piston system. This apparatus is uniquely capable of both PPV and NPV for rigorous same-specimen statistical comparisons and minimizes confounding factors. In PPV, air was pushed into the lung directly via a trachea-connected tube; in NPV, the tank volume was increased for air to be drawn into the lung; for both ventilation schemes, the resulting compressed lung pressure and volume were measured by the dual-piston apparatus^6,15^. An in-depth account of this system’s functionality is previously described^18^. From this joint ventilation-DIC methodology, the continuous global pressure-volume behavior and associated local surface strain measures were ascertained.

The test protocol included a 5 cmH_2_O preload, three inflation-deflation preconditioning cycles^81^,^34^, and a fourth test cycle wherein inflation was recorded via DIC^13,35^. This was implemented for PPV, followed by NPV at a matched lung volume ± 10%^6,15^, where the sequential order of ventilation mode has not been found to affect the lung response^36^. The sequence was conducted using 0.3 ml followed by 0.7 ml applied peak volumes, at 10 breaths per minute (BPM), thereby exploring quasi-static, murine lung capacities—from physiological tidal breathing, to near injurious limits, in accordance with past methods^6,37–41^. DIC parameters such as collection frequency, facet size, and post-processing methods were selected, as detailed previously^12,13^.

Principal major engineering strain and minor strains were obtained (GOM ARAMIS software), as well as the anisotropic ratio between them^21,42^. Additionally, shear strain was determined, where an overlaid coordinate system aligned with ventral-dorsal (VD, shortest axis) and cranial-caudal (CC, longest axis) lung axes, specified the directional strains^13,42^. Contour plots qualitatively depicted major strains at peak inflation (i.e., moment of maximum air delivery) to 0.7 ml for representative lungs. Histograms quantitively depicted the major, anisotropic, and shear strains at peak inflation (to 0.3 and 0.7 ml applied volumes). The mean and interquartile range (IQR; to assess heterogeneity^43^ of these strains were reported. A region of interest (ROI) analysis was performed by segmenting the lung DIC data along the previously defined VD and CC directions, for nine ROIs per specimens’ left lung (LL), superior lobe (SL), and inferior lobes (IL)^13^, uniformly applied to all lung specimens. Box-and-whisker plots quantified the mean major strains within these ROIs at peak inflation.

Continuous pressure-strain curves (i.e., average major strain versus lung response pressure) were evaluated as the local analog of pressure-volume (PV) curves for 0.7 ml applied volume^12^. Major strain histograms were correlated to these curves at select applied inflation volumes with additional surface strain evolutionary behavior detailed using the mean and IQR. Lung compliance slopes were computed via linear regression, and these bilinear curves often required two best-fit lines for R^2^ ≥ 0.95, consistent with classic PV curves^13,35,44–46^. The bilinear regimes were described by C_initial_ and C_final_, denoting the initial (gradual) and final (steeper) compliance slopes. Additionally, the lung pressure at the transition between the C_initial_ and C_final_ slopes was computed^46,47^. Finally, major strain values at each discrete DIC-generated point were divided by the global lung pressure to determine local-distensibility values and generate color maps. This measure was assessed for 0.2 ml, 0.5 ml, and the final peak 0.7 ml inflation volume to depict the spatiotemporal progression of lung compliance; because global pressures differ slightly between modes, these maps were intended to highlight spatial organization and regional heterogeneity rather than to compare absolute magnitudes between PPV and NPV. All calculations were performed using MATLAB (MathWorks Inc., Natick, MA, USA).

Statistical analysis was implemented with significance designated as *p < 0.05, **p < 0.01, ***p < 0.001 (GraphPad Prism 9 for Windows, San Diego, CA, USA). A paired two tailed t-test was used to compare PPV versus NPV strain metrics, in congruence with previous methods^33,48,49^. Supplemental analysis included an unpaired t-test to assess control versus exposed comparisons (within fibrotic and emphysematous groups).

## Results

**Fig. 2:**
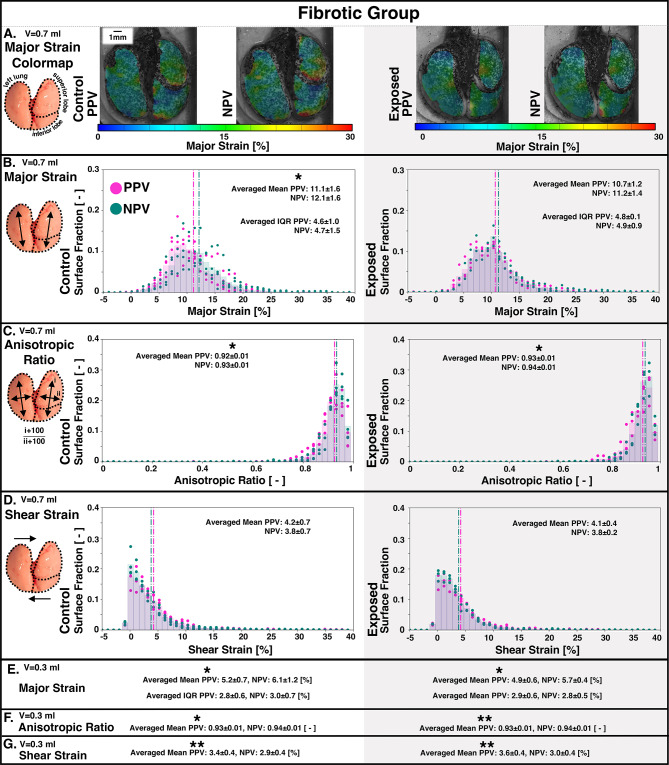
Fibrotic group lung strains yielded from PPV and NPV modes. (A) Major strain contour plots were shown at peak inflation for 0.7 ml applied volume. Histograms of specimens’ individual (dots) and averaged (bars) strain results were shown for (B) major strain values, (C) anisotropic ratio, and (D) shear strains. Specimen-averaged mean (dotted vertical lines) and IQR for these different strains were listed, and statistically significant PPV versus NPV differences were denoted with asterisks. Similarly, major strain, anisotropic ratio, and shear strain were shown in condensed version for 0.3 ml (E–G).

The fibrotic group (control and exposed) peak lung inflation strains under PPV and NPV were demonstrated in Fig. 2. Major strain contour plots for representative specimens at 0.7 ml applied volume qualitatively showed that NPV lungs had more high-strain regions (red) than PPV, particularly in the lower regions of the SL and IL (Fig. 2A). Histograms represented fibrotic lungs’ major strains, anisotropic ratio, and shear strains (for 0.7 ml; Fig. 2B–D). Mean major strain was significantly greater in NPV compared to PPV for controls (Fig. 2B). PPV lungs were more anisotropic (i.e., lower anisotropic ratio) compared to NPV for both control and exposed groups (Fig. 2C).

Figure 2 E-G reported the mean major strain, anisotropic ratio, and shear strains for fibrotic group lungs inflated to the lower applied volume of 0.3 ml. NPV mean major strain was significantly greater compared to PPV for both control and exposed specimens (Fig. 2E). Additionally, for both control and exposed groups, PPV anisotropic ratio was significantly lower (indicating more anisotropy) than NPV (Fig. 2F), and PPV shear strain was significantly greater than NPV (Fig. 2G).

**Fig. 3:**
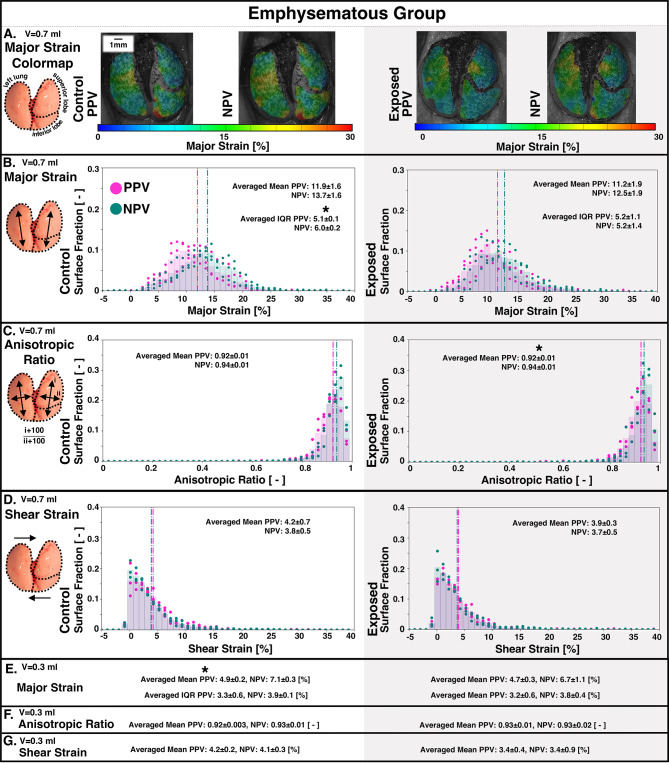
Emphysematous group lungs’ major, anisotropic, and shear strains arising from PPV and NPV at peak inflation to applied volumes of 0.7 ml (A–D) and 0.3 ml (E–G), with PPV versus NPV significances denoted with black asterisks.

Figure 3 demonstrated PPV and NPV peak lung inflation strains for the emphysematous group (control and exposed). Major strain IQR was significantly greater in NPV compared to PPV for control specimens at 0.7 ml (Fig. 3B). PPV compared to NPV had significantly lower anisotropic ratio for exposed group lungs at 0.7 ml (Fig. 3C). Mean major strain was significantly greater in NPV compared to PPV for control lungs at 0.3 ml (Fig. 3E).

Additionally, control versus exposed comparisons revealed shear at 0.3 ml was significantly greater for emphysematous control specimens compared to exposed in PPV (*p < 0.05; Fig. 3G).

**Fig. 4:**
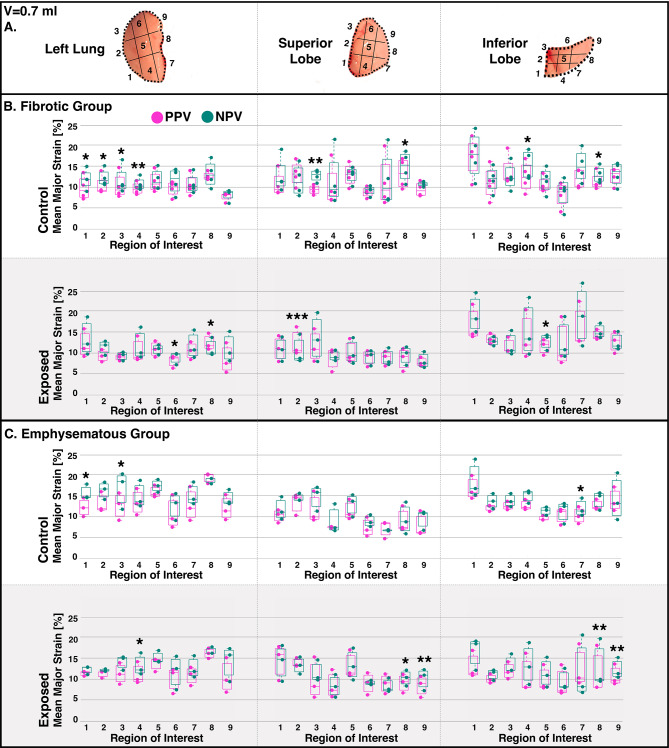
LL, SL, and IL regions were sectioned as denoted in (A) to generate box-and-whisker plots of ROIs’ mean major strain during PPV and NPV inflation to 0.7 ml peak applied volume, for (B) fibrotic and (C) emphysematous groups (individual specimens represented as dots). Statistically significant PPV versus NPV differences were indicated with asterisks.

Figure 4 illustrated the mean major strain within ROIs of each lobe at peak inflation (0.7 ml). NPV often showed greater strains within ROIs compared to PPV (significant differences shown; black asterisks).

Figure 4. LL, SL, and IL regions were sectioned as denoted in (A) to generate box-and-whisker plots of ROIs’ mean major strain during PPV and NPV inflation to 0.7 ml peak applied volume, for (B) fibrotic and (C) emphysematous groups (individual specimens represented as dots). Statistically significant PPV versus NPV differences were indicated with asterisks.

**Fig. 5:**
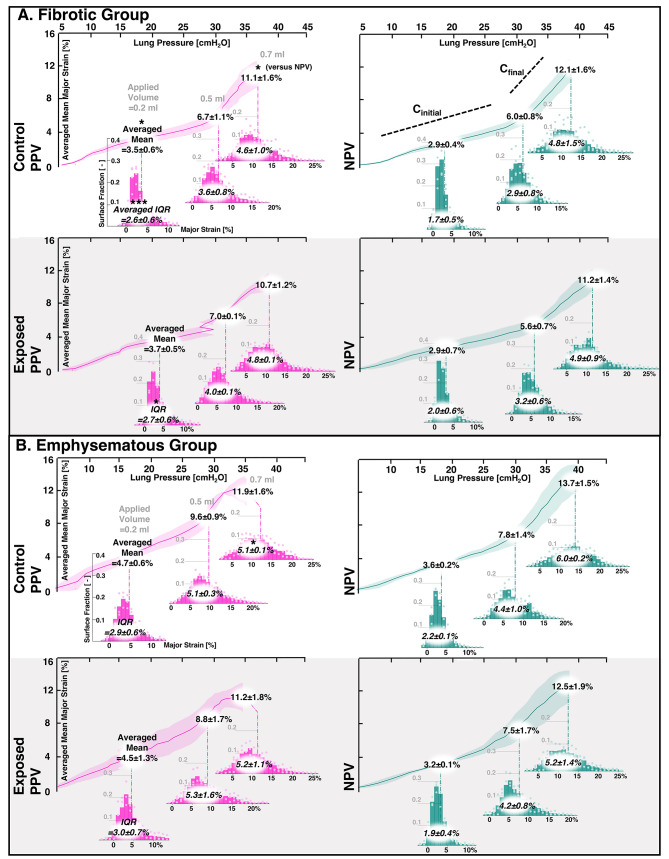
Pressure-strain curves were obtained for the PPV and NPV methods for inflation to 0.7 ml applied volumes. These curves were averaged between specimens (± standard deviation shadows) within the (A) fibrotic and (B) emphysematous control and exposed groups. Histograms of the major strain values at select volumes of 0.2, 0.5, and 0.7 ml were denoted (averaged mean and IQR shown). Significant PPV versus NPV differences were denoted with black asterisks.

Pressure-strain curves (i.e., the continuous relationship between global response pressure and local DIC major strains during ventilation to 0.7 ml) were shown in Fig. 5. Table 1 listed the associated metrics (C_initial_, C_final_, and slope transition). Resultant PPV curves did not consistently yield bilinearity, thus, C_final_ and transitions were incalculable and only NPV values were listed. PPV curves were also privy to abrupt pressure jumps or popping^35^, whereas NPV had a more gradual pressure-strain profile and consistent bilinearity (Fig. 5), with a tendency toward lower compliance values and higher transition pressures than PPV (Table 1). Histograms correlated to the pressure-strain curves at distinct applied volumes during inflation showed that earlier along the curve, volumes of 0.2 and 0.5 ml elicited greater mean and IQR values in PPV compared to NPV. However, when lungs reached the peak of inflation at applied volume of 0.7 ml, NPV tended to show greater mean and IQR values than PPV (Fig. 5). Control versus exposed comparisons (not shown) revealed fibrotic exposed specimens had higher intersection point values in NPV than controls (*p < 0.05).

**Fig. 6:**
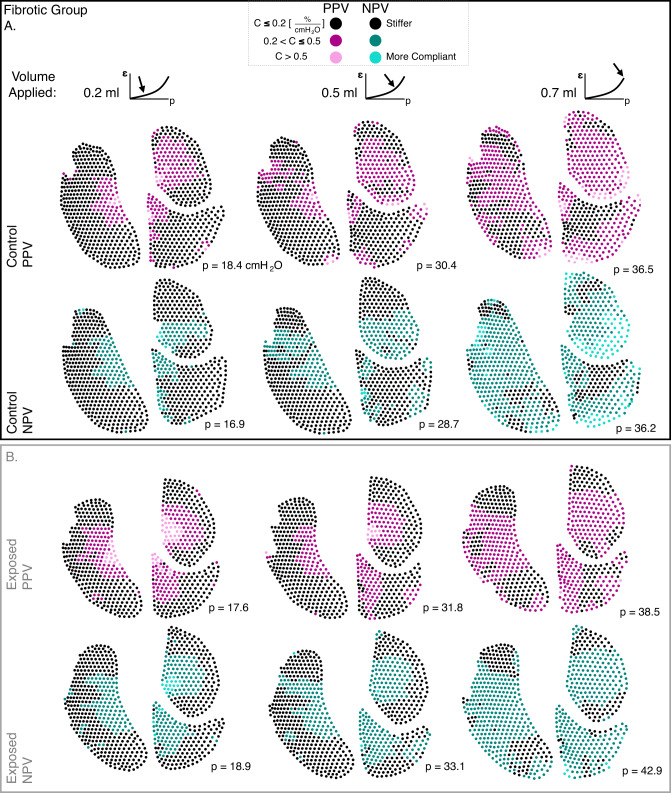
Fibrotic group (A) control and (B) exposed lung color maps of local-distensibility for representative murine lungs ventilated via PPV and NPV. Shown at select applied volumes of 0.2, 0.5, and 0.7 ml (peak inflation) during continuous ventilation, with global lung pressures indicated next to each specimen.

Figure 6 showed color maps of fibrotic group representative lungs’ local-distensibility values at each DIC-generated point, for progressive applied volumes of 0.2 and 0.5 ml during inflation to 0.7 ml. Lungs were generally stiffer (i.e., dark-colored regions) at 0.2 ml, while at 0.7 ml, lungs became more compliant (lighter-colored regions)—particularly for the NPV-inflated control lung (Figure 6A). Compliant regions tended to cluster in the lung periphery, especially for NPV.

**Table 1.**
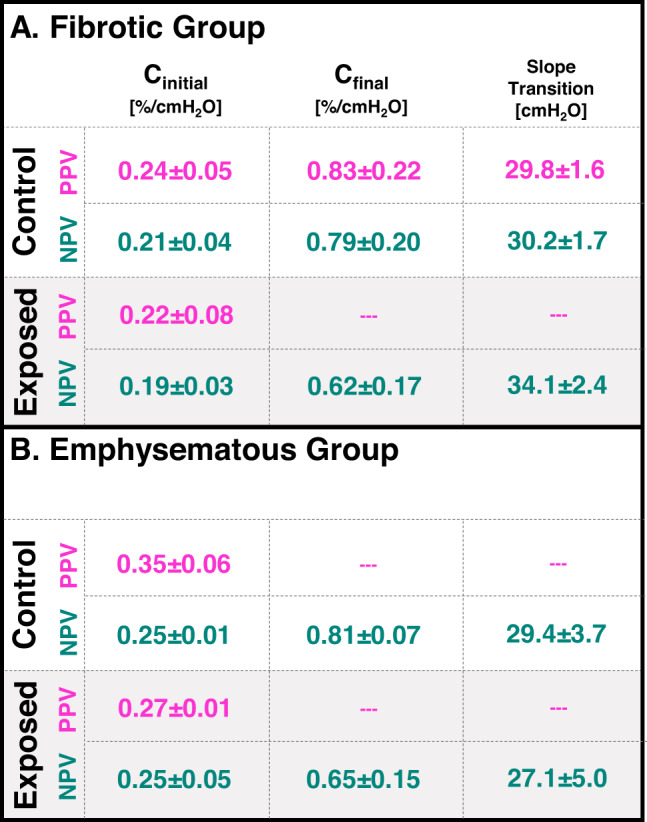
Quantified compliance and slope transition metrics associated with the pressure-strain curves of Figure 5.

**Fig. 7:**
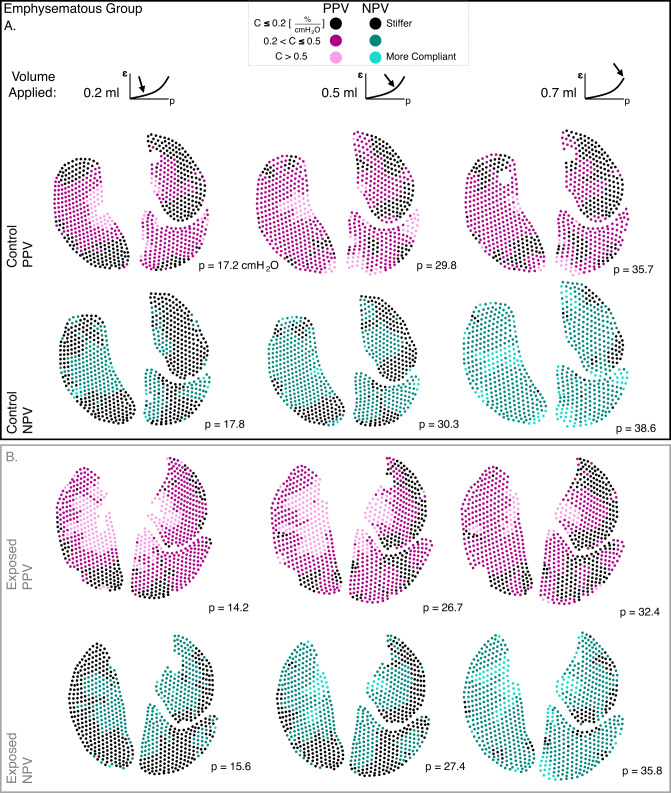
Emphysematous group (A) control and (B) exposed lung specimens’ local-distensibility color maps during PPV and NPV inflation.

The emphysematous group’s local-distensibility was shown in Fig. 7. PPV lungs initially showed high compliance regions at 0.2 and 0.5 ml (for both control and exposed lungs), but often became stiffer (lower compliance) at 0.7 ml, particularly for exposed lungs. NPV lungs, instead, showed a more gradual progression from low- to high-compliance values during increasing inflation. Emphysematous group control and exposed lungs (Fig. 7) were overall more compliant than that of the fibrotic group (Fig. 6).

## Discussion

Previous studies lack crucial explorations of PPV versus NPV differences for lungs in pathological states, where diseases exacerbate injury and therefore may exaggerate differences between the two strategies^5,7,15^. Here, we observe key interdependencies between pathological state and ventilation mode. Notably, PPV elicits high centrally located strains particularly in emphysematous lungs compared to NPV; moreover, greater strain heterogeneity, anisotropy, and shear is observed for PPV compared NPV, particularly for fibrotic lungs.

The spatial distribution of mechanical properties (e.g., strain) between regions is a key metric used to assess lung heterogeneity and, therefore, potential for injury. While the lung naturally exhibits heterogeneous distributions, previous studies link elevated levels of strain heterogeneity to disease, damage, and VILI^4,9–11,50^. For instance, in this current study, the IQR of strain values (measure of spread) tends to be greater in disease-exposed compared to control lungs (Fig. 5), particularly for the fibrotic group (Fig. 2B). This is in agreement with previous studies, which describe the fibrotic lung like a squishy-ball-in-a-net—with regional collagen deposition eliciting imbalanced under- and over-inflated damaging regions^51^. Thus, we exhibit how measures of heterogeneity are linked to potential lung damage.

In this current assessment of PPV and NPV, measurement of lung heterogeneity is used to compare the ventilation modes: notably, PPV shows increased IQR and therefore more strain heterogeneity compared to NPV—specifically at earlier 0.2 and 0.5 ml inflation volumes (Fig. 5). Thus, PPV appears to be linked with increased heterogeneity during progressive inflation compared to NPV, likely attributable to the way underlying airways are recruited between the two modes. Recruitment is region- and time-dependent: airways in peripheral regions—which are not engaged until the secondary recruitment stage (denoted by C_final_)—are notably shown to require more time to open in PPV^6,15,36^. These differences may in part stem from the distinct airway closure and reopening pressures between the two modes. PPV tends to involve more heterogeneous and higher local opening pressures, whereas NPV transmits the negative pleural pressure more uniformly, promoting earlier and more synchronous recruitment across regions^5^. Therefore, peripheral airways may not be recruited fast enough in PPV to accommodate the delivered volume of air, requiring central and upper lung regions to overcompensate; this behavior likely led to the greater heterogeneity observed here for PPV (at lower volumes), and such heterogeneity may indicate potential for VILI^3,5,13,44,52,53^.

Central overdistensions are indeed witnessed here, where PPV yields highest local-distensibility values in central regions, notably at the lower 0.2 ml volume (Figs. 6 and 7). Overall, this high compliance at the start of inflation likely arises from more aggressive initial recruitment in PPV, observed with the higher C_initial_ (compared to NPV; Fig. 5; Table 1). In fact, PPV often does not even elicit a distinct secondary recruitment C_final_ phase (Fig. 5; Table 1)—and this lack of secondary, peripheral engagement is likely why PPV lung peripheral compliances are lower than NPV once the lung is fully inflated (0.7 ml; Figs. 6 and 7). Comparatively, NPV appears to distend the lung more gradually (with a lower C_initial_; Fig. 5; Table 1), recruit additional alveoli (with consistent curve bilinearity from C_initial_ to C_final_; Fig. 5; Table 1), and show greater peripheral compliance (0.7 ml; Figs. 6 and 7)—suggesting its successful engagement of peripheral airways throughout the inflation process to more effectively oxygenate the lung, in agreement with previous literature^7,36,52,54^.

### Pathology and ventilation mode.

The heterogeneity imposed by PPV may also compound the negative effects of regionally-damaging pulmonary diseases for lungs under mechanical ventilation. For instance, PPV-inflated, diseased (i.e., exposed) lungs here produce overall greatest IQR values than controls or NPV-inflated counterparts (0.2 and 0.5 ml volumes; Fig. 5), indicating that diseased lungs under PPV-inflation are most heterogenous. In particular, emphysema may be impacted by the PPV-imposed central overdistensions discussed earlier: emphysema is a softening, overcompliant disease^17,30,55–57^, and prior studies show centrilobular regions are particularly predisposed to alveolar breakdown and weakening for elastase-exposed emphysematous mice^58^. Thus, these emphysematous lungs may be even more susceptible to mid-zone overdistensions when combined with PPV and its central overinflation. Evidence of this is observed, as the representative emphysematous exposed specimen yields highest local-distensibility values, manifesting in central regions, when combined with PPV inflation (0.2 and 0.5 ml volumes; Fig. 7B). Overall, this demonstrates how the interplay between ventilation mode and disease effects on the lung must be considered^15^.

### Collateral ventilation.

Specific modalities toward better oxygenation in NPV may include increased collateral ventilation: a mechanism which has been shown to improve ventilation-perfusion balance^59^. Murine collateral ventilation can be described by “popping” open of lung compartments mid-inspiration^60^. We further defined this behavior via strain measurements in previous work: the murine lung periphery pops open around mid-inflation, consequently eliciting high peripheral strains through peak inflation^13^. This current murine study again demonstrates this, and red (high strain) peripheral regions can be found in contour plots of Figs. 2A and 3A. While we find similar indications of collateral ventilation popping for both ventilatory modes, it appears to be more substantial for NPV compared to PPV. For instance, ROI comparisons show peripheral regions (e.g., ROIs 1, 4, and 7 of each lobe) often possess significantly higher strains in NPV than PPV (Fig. 4). This may explain the higher mean strain (i.e., average of all regions, including periphery) at peak inflation in NPV compared to PPV observed here for mice in Figs. 2B and 3B, (but not previously observed in pigs with their lack of collateral ventilation^5^. Though it should be acknowledged that while the higher peak inflation strains of NPV may be an indication of more collateral recruitment—to better distribute the air load and more effectively oxygenate the lung—these strains may still initiate damage, no matter their origin. The large, peak inflation air volumes sustained here approach murine total lung capacity, and may lead to regional overdistensions^61^—which is deleterious no matter the ventilation mode. We emphasize that the key finding here may be how strain is measured to be lower in NPV compared to PPV before peak inflation, in line with other studies which describe how NPV’s benefits are most achieved at low to mid-inspiration volumes^6,52^.

### Anisotropy

The anisotropic stretch ratio assesses potential distortions in expansion^13,62^. Here, the DIC surface strain measures may primarily represent properties of the top surface tissues: visceral pleura and parenchyma, which are generally regarded as isotropic^63,64^. Accordingly, ratios here are found to be near one (Figs. 2 and 3), demonstrating relatively isotropic expansion. Nevertheless, we find significantly more anisotropy in PPV compared to NPV (i.e., lower ratios; Figs. 2C, amp and F and 3C), in agreement with previous work^5^. Anisotropy has previously been used to assess progressing disease and injury, which alter lung function^13,62,65^—here, we find that PPV may impose a distorting effect on the lung, given the greater anisotropy. Furthermore, Fig. 2 (C, F) shows this PPV-anisotropy is most consistent amongst the fibrotic group exposed (diseased) and control (i.e., more mature compared to emphysema-control group) lungs. This may occur because fibrotic-disease, as well as maturation, are linked to changes in lung collagen fiber arrangement and proportion, and such structural alterations may encourage more distortion under PPV^57,66,67^.

### Shear strain

Generally, we find shear strains tend to be greater in PPV compared to NPV lungs (Figs. 2D, amp and G and 3D, amp and G). Previous studies associate lung shear strains to cyclic alveolar dis- and re-engagement^50,68^. Thus, the higher shear in PPV (measured here after multiple preconditioning cycles) may suggest more atelectatic, collapsed regions are present, which require re-engagement, further demonstrating the localized imbalances and insufficient recruitment mechanisms of PPV.

### Comparison with the existing PPV/NPV literature

It is well-established that if the lung were a simple elastic structure (e.g., balloon), its inflation would be governed by the transpulmonary pressure during ventilation and not the method whereby that pressure differential is created (i.e., via PPV or NPV)^5,36,80^. However, the lung is a complex viscoelastic organ—thus, differences in its response to the two ventilation modes are plausible and noted in previous work^6,15,69^. Nevertheless, PPV versus NPV differences are elusive, since traditional studies implementing global measures often do not find notable differences between the two modes’ effect on bulk lung behavior (given a matched volume history^33)^. On the other hand, examination of the lung using direct comparative PPV/NPV apparatuses in addition to a more granular level with the local strain response exposes disparities between the two ventilation modes^5,36^. Here, we find regional tissue-level differences between PPV and NPV: most notably, NPV yields significantly lower mean (major) strain than PPV at 0.2 and 0.5 ml (Fig. 5), suggesting reduced stretch and less potential for VILI at these volumes^3,5^. However, at 0.7 ml (peak inflation), NPV strains are greater (Figs. 2B and 3B). Interestingly, a previous PPV/NPV DIC study using porcine specimens (healthy), instead found strains at peak inflation were greater for PPV rather than NPV^5^. Thus, while both this current murine study and the previous porcine study show the lung strain response differs between PPV and NPV, the exact response is not necessarily analogous^70^; discrepancies may occur due to the disparate physiologies of the species used. For instance, mice, employed in this study, experience collateral ventilation near total lung capacity via the Pores of Kohn (as do humans)^60,71,72^, while pigs do not^73^. Such mechanisms determine oxygenation, recruitment, and lung expansion, and therefore, influence the overall strain deformation response; moreover, these recruitment mechanisms can be ventilation mode dependent^6,7,70^. Thus, these specific mechanistic features of lungs between species may drive an overall different PPV versus NPV strain response. Such considerations are noteworthy when selecting animals for human-translational clinical research^70,74–76^.

## Limitations and future directions

Though the native lung structure is preserved, extracting the organ from the chest cavity may limit findings and physiological relevance. For instance, previous studies speculate that the chest wall may exacerbate the overdistensions imposed by PPV; where chest wall resistance to expansion causes compression on collapsed alveoli such that they remain unengaged, while open alveoli receive the volume load to overdistend. NPV, on the other hand, directly expands the chest wall, and adjacent alveoli open more readily such that more units can accept the volume load^33^. Thus, our findings of overdistended central regions in PPV may be even more intensified in the presence of the chest wall; however, the murine chest wall is known for its softness, and therefore, this effect may be minimal^56,77^. Future aims include coupling a transparent chest wall (akin to recent novel techniques in the literature^78)^ with the spatiotemporal insights offered by DIC methods to further bolster potential insights.

Additionally, DIC provides measurements at the surface rather than deep lung strains, which is an inherent tradeoff for the valuable real-time, high resolution benefits of this strain quantification method. As such, direct observations on deep lung behavior are not possible in this current study. However, murine lungs are known to have deep lung structures (such a greater fraction of alveoli) nearer the pleural surface compared to other species, allowing more accurate predictions on how internal behaviors influence the topological surface strains^79^. Future work will aim to define the correlation between internal to topological strain measurements to address this limitation.

As with any animal model, use of murine specimens poses certain limitations. For instance, murine lung peak resultant pressure values are not matchable between PPV and NPV at the physiological volumes imposed here, but are so for other species (i.e., pigs)^70^; thus, we assessed volume- rather than pressure-matched PPV versus NPV data. While this facilitates a new perspective, it may produce differences between findings across species (i.e., porcine lungs which are pressure-matched between PPV and NPV may elicit dissimilar findings compared to murine lungs which are volume-matched). Even so, mice studies are the cornerstone of research advancements. For example, mice are central to studying induced diseases in a controlled manner to limit confounding factors and increase study robustness^30,74^. To that end, future work will include further PPV versus NPV characterizations for other murine induced diseased states. Of course, ultimate aims include carrying out these analyses in human donor lungs for greatest clinical impact.

## Conclusion

In this study, we explore how murine lungs with induced pathologies respond to PPV versus NPV via analysis of local DIC strain mechanics, revealing insights which global measures alone cannot afford. PPV generally exhibits greater heterogeneous, anisotropic, and shearing strains compared to NPV, particularly for fibrotic lungs—demonstrating how impacts of disease may be exacerbated by ventilation mode. Moreover, central overdistensions in emphysema are notably found to be amplified by the use of PPV. Overall, NPV appears to be most beneficial in reducing deleterious mechanical strains during the inflation process, but also shows more recruitment at peak inflation which may indicate better oxygenation. Ultimately, exploring the strain response of diseased lungs under PPV versus NPV can directly demonstrate how VILI may arise for relevant lung cases which necessitate use of a clinical ventilator. Thus, the insights provided here can be used to inform clinical strategies, as well as future research and computational models, which aim to predict and understand lung behavior.

## Data Availability

Upon reasonable request, the raw data supporting the conclusions of this article can be made available by the corresponding author via the contact form at bmech.ucr.edu.
